# PERCEPTIONS ON A DIGITAL HEALTH PHYSICAL ACTIVITY PROGRAM IN OLDER FAMILY CARE PARTNERS OF PERSONS WITH HEART FAILURE

**DOI:** 10.1093/geroni/igad104.2525

**Published:** 2023-12-21

**Authors:** Dawon Baik

**Affiliations:** University of Colorado, Aurora, Colorado, United States

## Abstract

Many family care partners (FCPs) neglect managing their own health and wellness when providing care to others. Older FCPs are more vulnerable in managing their health because of physical and psychological distress, age-related health needs, and less social support. We developed a digital health physical activity (PA) program, TPA4You, to begin to address these issues. We elicited perceptions of the integrated components of TPA4You (i.e., Zoom PA with a health coach, Fitbit, motivational text messaging) among older FCPs of persons with heart failure (HF-FCPs) using scenario-based design methods. Participants were recruited at the University of Colorado Hospital between September and December 2022. Fifteen qualitative interviews were conducted. Mean age was 65.9±4.4 years, 73% female, 93% White, and 13/15 were spouses. The perceived value of TPA4You was reported as (1) convenience because travel not required, (2) increased motivation from being held accountable by the separate technology components, and (3) consideration of improving FCPs’ health and wellness. Challenges described were (1) scheduling, finding time dedicated to exercising with the health coach by Zoom and (2) feeling overwhelmed, integrating the technology components in the FCP’s life. The keys to motivating PA and exercise through TPA4You were (1) usefulness of content in text messages and a good relationship with the health coach, (2) encouragement provided by TPA4You engagement, and (3) desire to improve their health and quality of life. Future studies should conduct field-testing of TPA4You in older HF-FCPs by providing one-on-one, hands-on sessions using the integrated technology components to optimize usability and acceptability.

